# SmartLact8: A Bio-Inspired Robotic Breast Pump for Customized and Comfort Milk Expression

**DOI:** 10.3390/biomimetics8020190

**Published:** 2023-05-04

**Authors:** Yuying Li, Marlenne Valadez Lozano, David Peña, Ish Kumar Gulati, Lin Jiang

**Affiliations:** 1 Department of Mechanical Engineering, San José State University, San Jose, CA 95192, USA; yuying.li@sjsu.edu (Y.L.); david.pena@sjsu.edu (D.P.); 2 Department of Biomedical Engineering, San José State University, San Jose, CA 95192, USA; marlenne.valadezlozano@sjsu.edu; 3 College of Engineering, San José State University, San Jose, CA 95192, USA; ish.gulati@sjsu.edu

**Keywords:** breast pump, soft robotic, piezoelectric sensors, oral-feeding mechanism

## Abstract

According to the 2018 National Immunization Survey conducted by the Center for Disease Control and Prevention (CDC), 83.9% of the breastfeeding mothers in the United States have used a breast pump at least once. However, the majority of existing products use a vacuum-only mechanism to extract milk. This causes common breast injuries such as nipple soreness, breast-tissue damage, and lactation complications after pumping. The objective of this work was to develop a bio-inspired breast pump prototype, named as SmartLac8, that can mimic infant suckling patterns. The input vacuum pressure pattern and compression forces are inspired from term infants’ natural oral suckling dynamics captured in prior clinical experiments. Open-loop input–output data are used to perform system identification for two different pumping stages that facilitates controller design for closed-loop stability and control. A physical breast pump prototype with soft pneumatic actuators and custom piezoelectric sensors was successfully developed, calibrated, and tested in dry lab experiments. Compression and vacuum pressure dynamics were successfully coordinated to mimic the infant’s feeding mechanism. Experimental data on sucking frequency and pressure on the breast phantom were consistent with clinical findings.

·

## 1. Introduction

The well-known reason why mothers choose to supply human milk to their infants is because it provides unmatched nutrition and immune support for their infants. In spite of most mothers’ intention to breastfeed, the CDC1 statistics showed that 60% of them stop sooner due to hospital practices, work policies, and breast injuries [1,2]. Early termination is largely associated with neurological and physical health issues for both mother and child [3]. For example, infants with a cleft palate cannot create the pressure needed to suck milk from the nipple, preventing them from being breastfed, and infants with Down syndrome cannot control their oropharyngeal structure to latch on during breastfeeding. Breast engorgement and abscess, ductal blockage provide inadequate milk production and limited milk supply [4]. Due to these reasons, a growing number of breastfeeding mothers use an electric or manual pump to support breast milk for their babies.

Existing milk pumps [5,6] usual extract the milk with only vacuum pressure, which is not similar to the infant’s intra-oral movements. The actions of milk pumps and natural suckling have many differences; for example, the positive compression of the infant’s palate, jaw, and tongue movement, which are important in breastfeeding [7]. A review in 1988 [8] summarizes that factors that influence milk flow at a feed are not only from both the mother and her infant but also the specific interaction between them. To design a breast pump that works effectively, it is essential to mimic the sucking patterns and reflex milk ejection of infants, while including fully controllable alternations in oral dynamics.

While feeding at the breast, infants control milk expression by adjusting both the intra-oral vacuum and oral compression pressure on the nipple–areola complex [6,9]. An oscillatory movement from the upper palate (maxilla), tongue and jaw assembly (mandible) was observed in previous clinical studies [10,11]; it is also usually observed via nipple deformation in the oral cavity in in vivo ultrasound images. Weber et al. [12] noted the importance of taking into account both oral compression and vacuum pressure. Positive oral pressure, or normal force, exerted by the infant’s mouth on the breast has received limited attention in recent decades. Several pioneering experiments compared the amount of milk ejected by breastfeeding mothers using a breast pump that only used a vacuum with a pump that used both a vacuum and compression [13]. When the compression component was active (pressure amplitude controlled by the mother), milk flow began before the first milk reflex was active, and peaks in milk excretion occurred sooner and more frequently. Further experiments showed that when ductal pressure was not at its peak, combined suction and compression resulted in faster milk release from the breast; when ductal pressure was at its peak, suction alone removed milk faster [14]. Among all the studies mentioned, only one measured the maximum compression that mothers could tolerate, which was 35–40 kPa [13].

Clinical assessments of breastfeeding explored the mechanism of milk production [15], the rhythmic vacuum pressure for milk extraction [9], and the relationship between infants’ oral muscle movement and the milk ejection [10]. While intra-oral pressure could yield adequate volumes of milk for infant consumption, more recent work demonstrated that mouthing dynamics are as effective as a vacuum for milk removal [11,14]. In addition, some studies suggested that vacuum-induced repetitive trauma is a real and significant concern for breastfeeding women who use breast pumps, and that proper education and use of the breast pumps could help in minimizing the risk of this condition. Waller et al. [16] conducted clinical experiments with breastfeeding mothers to find the main reasons for the early failure of breastfeeding. They found that the frequency of high pumping pressure was attributed to certain anatomical defects of the nipples and terminal ducts, which were the main reason for early breast milk cessation. Kent et al. [17] examined the relationship between breast pump suction and the occurrence of nipple pain and trauma in lactating women. The authors found that higher suction pressure was associated with a greater incidence of nipple pain and trauma. To the best of our knowledge, the effect of oral interaction to the breast during pumping has not been considered. For a better pumping experience for mothers, and as a future smart breast pump that is comfortable and effective, a fully controlled bio-inspired breast pump that mimics oral muscle contractions needs to be designed and studied in vitro.

Although breast pumps inspired by the biomechanics of breastfeeding [18,19] and biomedical devices for training infant’s suckling [20,21] have been studied during the past few years, a breast pump device that could systematically mimic the infant’s oral mechanism on the breast and assist in milk expression has not been deployed. In addition, commercially available breast pumps exert much more pressure than that of an infant, and it is largely associated with breast injuries and early breastfeeding termination. In a study based on 1844 samples from mothers, about 62% reported pump-related problems, and 15% reported injuries after breast pumping [22]. The risks include nipple soreness, breast-tissue damage, and lactation complications. Table 1 compares the pressure from typical breast pumps with that from infants from clinical studies. Moreover, it is well established that decreasing vacuum pumps and increasing compression pressure on the breast will result in mothers being able to breastfeed more effectively and comfortably. A review in 1988 [8] summarized that factors influencing milk flow during feeding are not only from vacuum suction, but also from the specific interaction between the mother and her infant. This was then studied in the corresponding author’s preliminary work. The positive compression of the infant’s palate and jaw, and tongue movement, are both known to contribute to milk removal in breastfeeding [7]. A pioneering study conducted by Alekseev et al. found milk excretion peaks occurred more frequently and earlier during periods of compression [13]. In terms of vacuum suction, Ramsay et al. [5] found that the vacuum expression pattern did not change milk ejection patterns or the characteristics and efficiency of milk expression. The research of Kent et al. in 2006 [23], found that mothers’ maximal comfortable vacuum produced the maximal milk yield. Our ultimate goal was to produce a breast pump that could provide mothers with a safe, comfortable, and portable milk expression process. Moreover, the bio-inspired breast pump is expected to be reliable and sustainable in providing human milk to infants, and to supports their immune system development. Its broader impact is promoting the growth of the national immunization rate of infants and achieving the CDC’s Healthy People 2030 Breastfeeding Objective.

Followed by a preliminary clinical study [7] that successfully measured the oral vacuum pressure and captured the tongue movement using imaging techniques, the authors were able to identify the positive pressure or compression pressure that an infant would apply on the mother’s breast and use that data for the bio-inspired breast pump design. An innovative breast pump prototype developed mimics the infant suckling pattern during breastfeeding. With the fuzzy logic controlled soft pneumatic actuators and a vacuum pump, this prototype provides peripheral compression and oscillatory vacuum pressure that simulates the infants’ intra-oral movements during breastfeeding. The SmartLac8 breast pump could support breastfeeding mothers with a safe, comfortable, and portable milk expression process. An automatic control system, soft robotic actuators, and flexible sensory pads were designed and developed in the apparatus to mimic the breast–infant interaction mechanism during breastfeeding. All components in the apparatus can be modified and customized based on the pumping frequency and the pressure feedback from the mother’s breast, which assist in optimizing milk excretion with a comfort pumping experience.

## 2. Preliminary Data from Clinical Study

Fifteen mother–infant dyads were initially recruited through either the Australian Breastfeeding Association or community health centres in Western Australia approved by the Internal Review Board at The University of Texas at Dallas (IRB 16-41) and the Human Research Ethics Committee of The University of Western Australia as outlined in [7]. A total of eight dyads provided positive oral pressure data and negative vacuum data, with one dyad providing two sets of data. The ages of infants ranged from 6 days to 21 months. The ultrasound images and the vacuum pressure were obtained by an endocavity convex transducer (Acuson, XP10, Siemens, Mountain View, California, USA) and placed under the infant’s chin and a silicon vacuum tube (650×4mm) was connected to a disposable pressure transducer (Cobe Laboratories, Frenchs Forest, NSW2086, Australia) as outlined by Geddes et al. [29].

**Figure 1:**
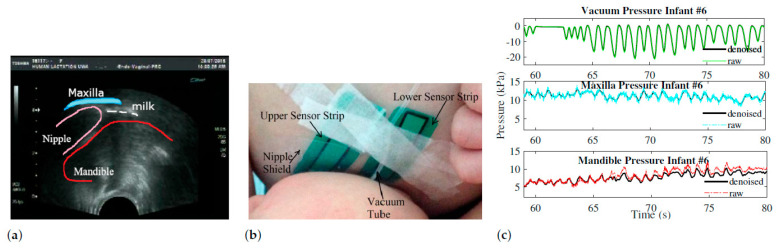
(**a**) Ultrasound imaging of a Term Normal Infant’s oral cavity during breastfeeding visualizes the structure of maxilla (upper hard palate), manidble (tongue and jaw), nipple, and milk flow; (**b**) Sensor placement during clinical study [30]; (**c**) Raw intra-oral pressure data for Infant #6.

Two flexible resistance pressure sensor strips 9801 and 9830 with the I-Scan System (Tekscan Inc. Boston, MA, USA) were attached to the breast and covered with a breast shield to minimize moisture exposure to the strips and to prevent the strips from entering the mouth of the infant (see Figure 1b). Ultrasound imaging was used to determine nutritive and non-nutritive suckling periods, as well as to visualize the changes in the nipple dimensions from infant pressures. Figure 1a shows a single image with the oral structure marked in the image from ultrasound videos of Infant #6 during their clinical experiment.

There was an oscillatory pattern for positive oral pressures in all infants, both in their maxilla (upper palate) and their mandible (tongue and lower jaw). During breastfeeding, total pressure was applied to the breast by the maxilla at 11.23 kPa and by the mandible at 5.65 kPa. The pattern of maxilla and mandible pressures match the vacuum peaks during these suck cycles. As a result of a local minimum vacuum (around −20 kPa), maxilla and mandible pressures reach a local minimum. It is evident in the ultrasound images that the infant’s mandible drops to create a vacuum as a result of this phenomenon. Therefore, infants use positive pressure on both sides of the areola to control milk extraction, and this bilateral pressure application should be considered when designing breast pumps.

## 3. Materials and Methods

**Figure 2:**
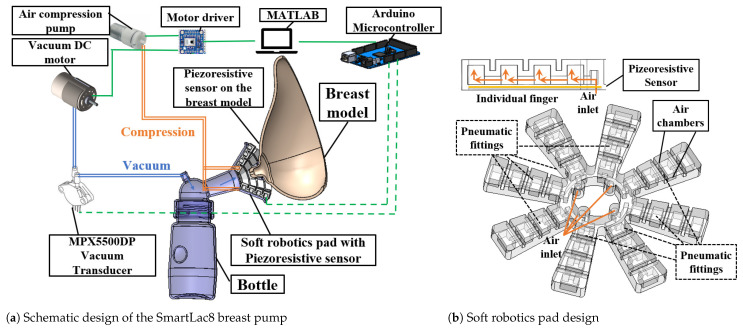
(**a**) Blueprint for the breast pump design, and (**b**) soft robotic pad model in SolidWorks.

To imitate the infant’s oral behaviour involving coordinated vacuum and compression pressure on the breast, a finger-like soft robotic pad consisting of eight pneumatic actuators was designed and applied on the breast pump. For capturing the air pressure data in the chamber, piezoelectric sensors were attached to the pneumatic actuators. Another set of piezoelectric sensors were fabricated on a circular pad and attached on the breast model as a nipple shield to capture pressure data. All components are inspired by the clinical observation of natural breastfeeding. The schematic configuration of the breast pump apparatus is shown in Figure 2a. The apparatus consists of the following:

- A flexible breast phantom for testing
- A vacuum pump that generates rhythmic intra-oral pressure
- Two miniature pneumatic pumps along with two solenoid valves that generate rhythmic compression pressure
- A soft robotic pad with eight pneumatic actuators for compressive-pressure mimicking
- A piezoelectric sensor pad for data capturing
- An inline pressure sensor that captures air flow rates
- Drivers and control systems for commanding the inputs and recording the output components.

### 3.1. Bio-Inspired Soft Robotic Pad for Breast Pumping

Figure 2b provides a schematic view of the designed fingers on the soft robotic pad. The eight finger soft robotic pad shape is inspired from breast massages for encouraging milk flow. The material we used is platinum silicone, which is a medically approved material that is semi translucent and safe for human interaction [31]. It is approved by regulatory agencies such as the U.S. Food and Drug Administration (FDA) for certain medical uses. Platinum silicone is often used in medical devices and implants because it is biocompatible, meaning that it does not cause a significant immune response or toxicity in the body [32]. It is also resistant to bacterial growth and is able to withstand high temperatures and pressures, making it suitable for use in preclinical design and fabrication stages [33]. Each pneumatic finger on the soft robotic pad considers the following design specifications presented in Table 2: elastomer wall thickness, air chamber size, contact area, shape, and size of the breast pump. Mosadegh et al. [34] and Sun et al. [35] correlated the various pneumatic actuator wall thickness with the force and pressure output using Eco-flex 00-30 silicone elastomer (Smooth-on Inc. Philadelphia, PA).This silicone shore hardness is 00-30. The wall thickness of the pneumatic pad was set to be 3 mm, and displacement was set to be 6 mm for the soft robotic pad. The pad requires about 30 kPa to fully actuate. Moreover, the force output ranged approximately from 0.5 to 2 N for 6 mm displacement during the actuation.

Estimated chamber size for the pneumatic actuators is based on a study by Sun et al. [35], which provided the foundation calculation for the force-displacement characteristics of bending-type soft pneumatic actuators. With the 30 kPa pressure that the air pump provides, the 3 mm wall thickness was selected with 6 mm displacement and 2 N of force. Furthermore, the individual chamber area was calculated as $\frac{2N}{30kPa}=0.000066$ m${}^{2}$. The chamber width and height for each pneumatic actuator finger was set to 5 mm. The estimated air chamber length was about 7.5 mm. There were four air chambers in each pneumatic fingers. Listed below are the features of the eight fingers soft robotic breast pump pad.

#### Fabrication of the Soft Robotic Pad

**Figure 3:**
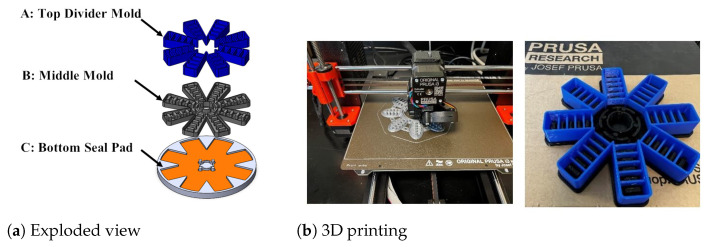
(**a**) Exploded view for soft robotic pad master moulds, and (**b**) 3D printing moulds with the PRUSA printer.

The SolidWorks exploded model view in Figure 3 displays the master moulds for fabrication. Three-layer moulds were designed and 3D printed based on the finalized dimensions to form the soft pneumatic actuator pad. The SolidWorks model .STL files were sent to the PRUSA slicer software to generate G code files. Then, the G code was loaded to the PRUSA M3 3D printer to generate the moulds. The moulds were printed with PETG material with a detail print setting, with a layer height set to 0.10 mm.

The fabrication process of soft robotic pads involves layering Ecoflex-0030, reinforcing the bottom seal, and embedding a total of eight piezoelectric sensors in the bottom seal. The fabrication process is explained in the following steps.

Step 1: Ease Release 200 is sprayed onto mould parts A, B, and C, as shown in Figure 3a. Next, the top and middle moulds are filled with Ecoflex, and overflow is prevented by filling gaps with clay. Then, the solution is poured onto the mould to form the actuator chambers.

**Figure 4:**
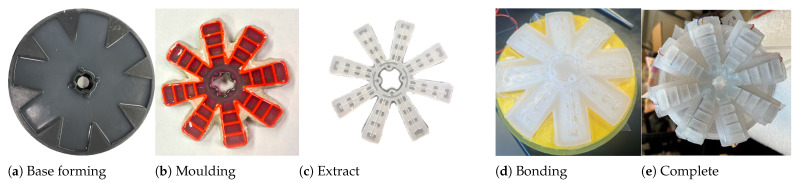
Fabrication process of the soft robotic pad with pneumatic fingers: (**a**) Pre-formed base seal for the pad; (**b**) mould for the pneumatic air chambers; (**c**) extracted model from the master mould; (**d**) bonded base to the pneumatic fingers; and (**e**) completed air chambers and pneumatic channels.

Step 2: Ecoflex silicone 00-30 solution A & B is mixed in a 1:1 ratio, the mixture is de-gas in a chamber, and poured onto the mould to form the actuator chambers, as displayed in Figure 4c.

Step 3: The base mould is formed with Ecoflex A-10 solution, measured and mixed, and poured onto the base plate, and de-gased.

Step 4: Both the soft robotic pad body and base are de-moulded from the moulds, visually inspected for bubbles or rough surfaces, and the custom-made piezoelectric sensor is placed on the base pad.

Step 5: The actuator pad body and base elastomer piece are bonded with an additional layer of Ecoflex 00-30 solution. The mix, de-gas and pour process is repeated to seal the gaps between pieces. Lastly, the pad is de-moulded as displayed in Figure 4e.

### 3.2. Sensor Fabrication

**Figure 5:**
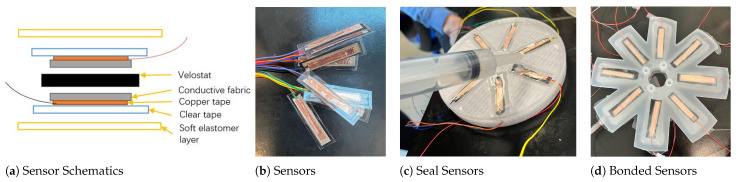
Fabrication process of the custom-made piezoelectric sensors: (**a**) Sensor layout for fabrication, (**b**) piezo-resistive sensors sealed with clear tape; (**c**) seal sensor on the base mould; and (**d**) bonded sensors to the base of the soft robotic pad.

A custom-made pressure sensor was developed to bond with the elastomer and be embedded into a soft pneumatic actuator. Each sensor consists of one sensing layer and two actuating layers. The actuating layer of the soft piezo-resistive sensor consists of two pieces of copper tape, two pieces of the conductive strip, and two strips of wire. The piezo-resistive layer (Velostats, Adafruit) is a conductive layer that changes its resistance value according to the amount of pressure acting on it. Its dimensions are 20 mm length by 5 mm width by 0.1 mm thickness. Conductive strips and copper tapes were used to improve the conductivity of the wires acting on the piezo-resistive layer. Furthermore, the finalized pressure sensors were sealed with clear tape. The final thickness of the custom-made piezo-resistive sensor was 0.3 mm. See the left of Figure 5a for a layer break-down, and the right for the sensors.

Next, these custom piezoelectric sensors was sealed into soft elastomer layers, which made bonding them to the soft robotic pads easier. To create flat elastomeric layers with flexible sensors, a 3D-printed mould was created with eight flexible pressure sensor slots on the front and a flat spindle cut-out on the back. The first step was to drop 1 mL of mixed Ecoflex 00-30 and de-gas the solution before applying to each slot. The second step was to spin the mould so that the elastomer layer is evenly distributed. The setup of the spinner was velocity (V) = 500 rpm; acceleration (A) = 50 rpm/s; time (T) = 5 s.

After spinning, we placed the mould onto the hot plate (set to 60 ${}^{\circ }$C) to speed up the curing process. When the base thin elastomer layers were cured, we placed the sensor in the slot and pour another 3 mL of mixed and de-gassed Elastomer 00-30 with a syringe, as shown in Figure 5c.

Next, the mould was placed inside the chamber to pull the vacuum pressure to −1 bar. This process helped force air between the sensor and elastomer layer. This way, the sensor lays flat and centred between the two elastomer layers. The elastomer was then placed on the hot plate to speed up the curing process or it was left at room temperature based on the suggested curing time. Final step was to remove the flexible pressure sensors from mould, one at a time.

### 3.3. Sensor Calibration

**Figure 6:**
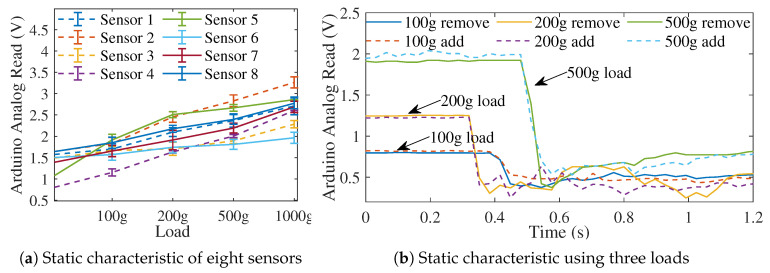
(**a**) Static characteristics using different weight loads, and (**b**) dynamic characteristic by applying pulse loads and testing the response delay of the sensor.

Static and dynamic characteristics [36] of the custom-made piezoelectric sensors were calibrated before the experiments. For sensor static characterization, increasing and decreasing calibration weight loads (0, 100, 200, 500, and 1000 g) were applied to the force sensors with a sensor contact area of 0.00042 m${}^{2}$, which provided a pressure range from 0 to 98 kPa. Accumulated data of static characterizations for both types of sensors are presented in Figure 6a. There was strong linearity among all sensors. A correlation function was used for each sensor and converted into Arduino analogue readings, which were then used as inputs to the control system.

For dynamic characterization, 100, 200, and 500 g weights were separately applied on the sensors and manually removed to test the sensor’s ability to respond to pressure changes. A response time was recorded when the pressure was instantly released. Figure 6b shows that the time required for the output to drop from 90% to 10% ranges from 0.03 to 0.07 s, less than the pressure profile updating rate at 0.1 s during experiments. These sensors proved to have sufficient dynamic characteristics for the measurement response purposes.

### 3.4. Complete Design, Control System and Experimental Setup

**Figure 7:**
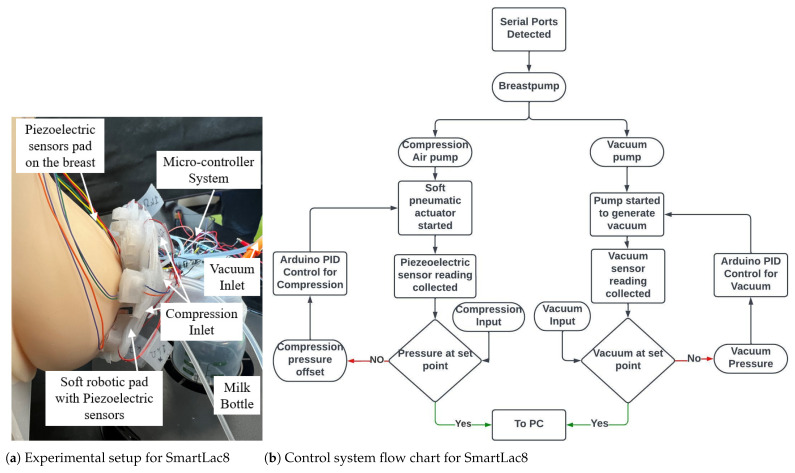
(**a**) Experimental setup includes a piezoelectric sensor pad on the breast with the same sensors for the soft robotic pad to capture pressure data on the breast; (**b**) flowchart for the constructed control system for the SmartLac8 breast pump.

The complete experimental setup for testing the bio-inspired soft robotic breast pump with a control system is presented in Figure 7. The flexible and human-tissue-mimicking breast phantom (Simulab, Seattle, WA, USA) was used for experimental study. The vacuum pump was pre-programmed to generated sinusoidal intra-oral pressure that follows the vacuum pressure profile of an infant based on clinical data [7]. A NXP pressure sensor (MPX5500, Digikey) was connected to the air loop to measure vacuum values in the tube. A simple closed-loop MIMO (multiple-input multiple-output) PID (proportional-integral-derivative) control strategy was designed to control all motors with one central control system. Feedback from the vacuum transducer and custom-made piezoelectric pressure sensor pads were captured and imported to the analogue reading pins on the micro-controller. Four piezoelectric sensors bonded on a silicone pad were used to measure the surface pressure from the nipple–areola area. Figure 7 shows the sensor locations on the breast phantom.

The real-time experimental control architecture is illustrated in Figure 7b. The PC first identified the number and location of serial ports that were connected to the micro-controller (Arduino Mega 2560, Arduino). Vacuum and compression pressure profiles were pre-imported to Arduino Mega 2560. A MATLAB program was employed to connect with the Arduino modules and process and transmit real-time data to the hardware.

For the bio-inspired robotic breast pump, all experiments employed a user-defined profile to showcase the successful replication of infant suckling behaviour. The vacuum frequency first started at 0.6 Hz, and then changed to 1.2 Hz. The local minimum and maximum vacuum for the first stage were −20 kPa and −8 kPa, respectively, whereas the local minimum and maximum vacuum for the second stage were −15 kPa and −5 kPa, respectively. Equations 1 and 2 demonstrate the pressure input profiles used in the experiment.

(1) $$\begin{array}{cc} \; & P_{vac.}=\left\{\begin{array}{ccc} 0 & 0\leq t<85\;s & \\ -5cos(2.4\pi t)-10 & 85\leq t<190\;s & Stage1\\ 0 & 190\leq t<230\;s & \\ -6cos(1.2\pi )-14 & 230\leq t<500\;s & Stage2\\ 0 & 500\leq t\leq 520\;s & \end{array}\right. \end{array}$$

(2) $$\begin{array}{cc} \; & P_{comp.}=\left\{\begin{array}{ccc} 0 & 0\leq t<85\;s & \\ 5cos(2.4\pi t)+10 & 85\leq t<190\;s & Stage1\\ 0 & 190\leq t<230\;s & \\ 6cos(1.2\pi )+14 & 230\leq t<500\;s & Stage2\\ 0 & 500\leq t\leq 520\;s & \end{array}\right. \end{array}$$

## 4. Results

Open-loop system identification experiment was conducted to extract the breast pump mechanical model using the sensor data. A two-staged experiment protocol was developed to test the setup’s feasibility. PID control parameters were selected based on the identified system and imported into the microcontroller unit. Tests were run utilizing the closed-loop control setup on the breast pump.

### 4.1. Soft Robotic Pad Actuation and Open-Loop System Identification

**Figure 8:**
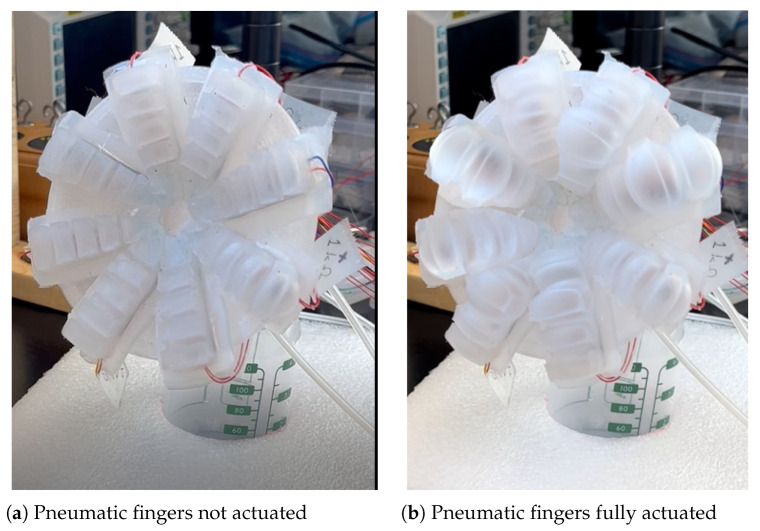
Experimental results on the soft robotic pad actuation with images for (**a**) not actuated pneumatic fingers, and (**b**) fully actuated pneumatic fingers.

Leakage checks were performed before each experiment. Figure 8 demonstrates the deformation on the soft robotic pad after being fully actuated by the air pump at 30 kPa. All eight pneumatic actuators were active. The chambers popped with pressure and provided compression to the mother’s breast. As shown in the Figure 8b, when popped, most deformed air chambers were in the middle part of the fingers (the second and third chambers). The contact area of the pneumatic finger and the breast was approximate 10–15 mm by 5–10 mm, comparable to the area of contact for an infant’s tongue when latched on to their mother’s breast based on a tongue dimension study in a 1985 [37].

The open-loop data acquisition was performed using Arduino and MATLAB code, the input voltage data in V and output pressure reading data in kPa were collected from the serial port communication between the PC and Arduino. The collected data included a lot of noise and uncertainties. We performed a system identification using offline open-loop system identification. All eight sensors worked and provided analogue data to the PC through Arduino serial port communication.

The soft robotic pad on the breast pump is a non-linear structural material. Furthermore, there are various sinusoidal stages of pressure inputs. Hence, we used the stochastic approximation algorithms [38] to obtain the unknowns for the non-linear system at each stage (two stages in this paper). The inputs were the vacuum pressure and compression pressure. The outputs were the vacuum transducer data in the air loop and the averaged sensor data from all eight sensors on the soft robotic pad. Further PID controllers for each stage were designed based on the identified system.

We used the recursive least squares (RLS) method for system identification [39]. The estimated model was constructed as a second-order system presented in Equation (3), where y(k) is the output of the system at time *k*, u(k) is the input to the system at time *k*, $a_{i}^{0}$ is the coefficient of the previous outputs in the system’s response, $b_{i}^{0}$ is the coefficient of the previous inputs in the system’s response, and v(k) is the measurement noise at time *k*. Model coefficients for the system in two different stages are demonstrated in Table 3. Data regarding system identification performance with RMSE and model fit are also presented in Table 3.

**Figure 9:**
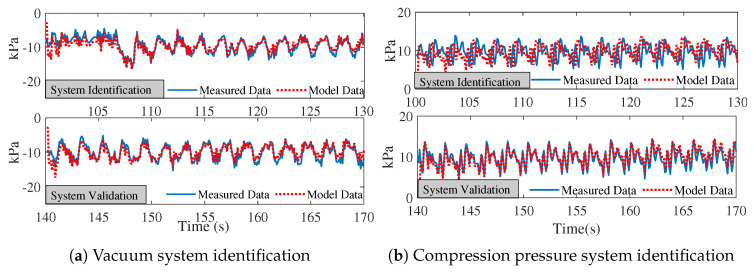
System identification for stage 1 pumping frequency and strength: (**a**) vacuum pressure system identification, and (**b**) compression pressure system identification.

Stage 1 active pumping ran from time =85 s to time =190 s. Data for system identification was from time =100 s to time =130 s, and data for system validation was obtained from time =140 s to time =170 s. Figure 9a,b demonstrate the system identification process and performance for vacuum and compression pressure. As shown in Table 4, the RMSE for stage 1 vacuum and compression was 2.3727 and 1.6509, respectively, indicating a goodness of fit during 100–130 s of pumping.

**Figure 10:**
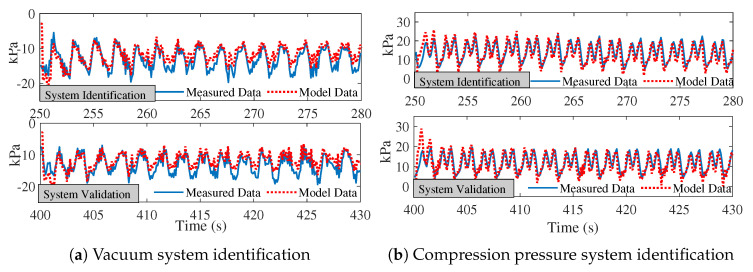
System identification for stage 1 pumping frequency and strength: (**a**) vacuum pressure system identification, and (**b**) compression pressure system identification.

Stage 2 active pumping ran from time =230 s to time =500 s. Data for system identification was from time =250 s to time =280 s, and data for system validation was extracted from time =400 s to time =430 s. Figure 10a,b demonstrate the system identification process and performance for vacuum and compression pressure. As shown in Table 4, the RMSE for stage 2 vacuum and compression was 3.6542 and 3.1405, respectively, indicating a goodness of fit during 140–170 s of pumping.

(3) $$y\left(k\right)=\Sigma_{i=1}^{n}a_{i}^{0}y\left(k-i\right)+\Sigma_{i=1}^{n}b_{i}^{0}u\left(k-i\right)+v\left(k\right)$$

### 4.2. Closed-Loop Controller Design and System Performance

The mechatronic system for the SmartLac8 system enabled feedback controls for two air pump motors for soft robotic pad actuation and one vacuum pump for suction using the piezo-resistive sensor data from the soft robotic pad and the piezo-resistive sensor on the breast phantom. A closed-loop feedback control system running on a micro-controller provided the real-time robust coordination of the soft robotic system. Arduino interacted with the vacuum and compression pump motors through the MATLAB simulink using the tuned PID parameters following system identification. The control system was designed with two levels. The first level collected control feedback signals from the motor encoder, pressure sensors and vacuum pressure transducer. In the second level, real-time set points were tracked from the system input using the tuned PID parameters on the Arduino board.

**Figure 11:**
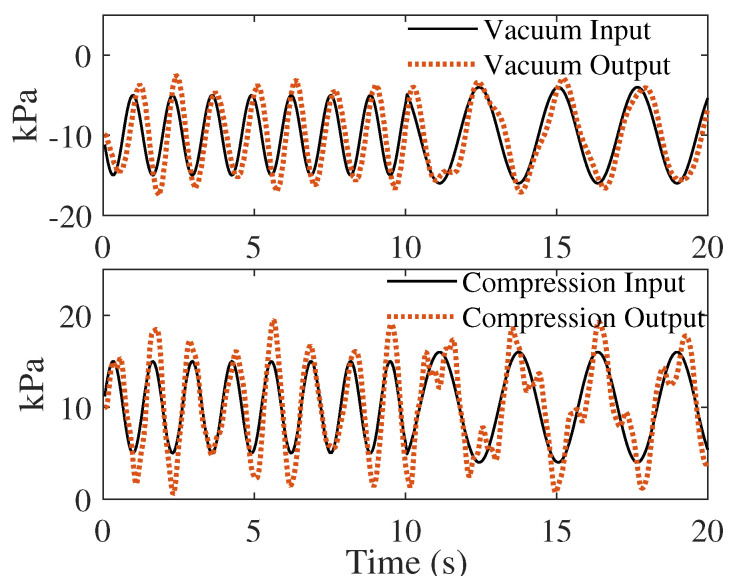
Tracking performance for the real-time PID control vacuum and compression pressures.

Figure 11 shows the dynamic-pressure-tracking performance generated by the oral breast suckling simulator under various pressure frequency. Table 4 lists the PID parameters, response time and tracking error for the vacuum pump control and compression air pump control. Within 0.7 s and 0.8 s after frequency variances for the vacuum and compression pressures, respectively, the tracking error decreased to ±10%. No significant magnitude changes were observed, and the frequency change corresponded to the input pressure dynamics. Although vibrations and environmental errors still affected the results of real-time control, both pumping systems were stable after onboard PID tuning. Hence, the breast pump achieved the desired frequency tracking in one suck cycle where the frequency ranged from 0.6 to 1.2 Hz in two stages. The average compression pressure on the breast was 12.25±5.42 kPa. These pressure results are consistent with the clinical observations of infant suckling patterns during breastfeeding [7].

## 5. Discussion

This work designed and developed a bio-inspired breast pump with a soft robotic pad using pneumatic actuators to mimic infant compression forces on the mother’s breast during pumping. The geometry was inspired by infant oral muscle dynamics and feeding physiology following a clinical study by the authors [7]. The designed architecture contains a soft robotic pad with eight finger pneumatic actuators controlled individually with air pumps, linear motors for vacuum and air pumping, custom-made piezo-resistive sensor pads, digital pressure transducers, and onboard feedback controllers with a proportional-integration-derivative (PID) control algorithm. In particular, the MATLAB program linked with Arduino hardware was developed in this work for real-time data monitoring and feedback control. We performed dry laboratory tests on the reliability and robustness of the breast pump. Our results confirmed the better performance with the feedback control loops for vacuum pressure tracking and non-linear soft robotic control. The bio-inspired breast pump, i.e., SmartLac8, successfully mimicked infant oral suckling behaviour with a better fidelity.

Possible limitations in this preliminary design may due to the offline system identification and the simplification of the environmental error. The onboard PID control was based on the transfer function of the motors, generated by open-loop system identification. Advanced real-time PID control will be more precise in tracking the vacuum and compression pressures, but will also increase the computational cost. The current maximum percentage error in the simulator was <10%, acceptable as a preliminary device for feasibility tests. Future work on this simulator includes a flow experiment for pump efficiency, and applying an advanced level of control algorithms to process the data in real time. It will also help in the study of fluid–structure interactions using soft actuators.

Despite these challenges, this work provides a customized, reproducible, and accessible robust breast pump for mothers for comfortable breast pumping. The SmartLac8 breast pump is slightly more expensive because of its additional control system for smart pressure control, but we anticipate a considerable acceptance among mothers since it reduces vacuum usage and mimics infant suckling in a natural manner. All electronics are actuated under 5 V, making it safe, portable and convenient to use. Integrated with an advanced control unit, the breast pump is the first fully controllable device that replicates the biomechanics of breastfeeding and functions as an educational, training, and research tool. We can also tune the compression forces based on varying the vacuum frequency and strength to match with the physiological mechanics of an infant’s oral suckling during breastfeeding and provide mothers both comfort and emotional support. While pumping, mothers can input and change their desired pressure for vacuum suction and compression pressure. Furthermore, it is notable that the design robustness of the SmartLac8 breast pump allows for the commercialization of a personalized, easy-to-use, reproducible advance robotic pump for mothers, as it can provide a controllable and tolerable pressure range due to the effective vacuum and compression pressure division and coordination.

## 6. Conclusions

A physical breast pump prototype with soft pneumatic actuators and custom-made piezoelectric sensors was designed, fabricated, and tested. Moreover, an onboard feedback control system based on pressure readings from the breast phantom was implemented into the breast pump prototype. The developed user-friendly and portable breast pump included multiple sensors to measure and monitor the pressure levels, allowing the user to tune the device during pumping. The proportional-integration-derivative (PID) controller implemented in the breast pump design successfully provided stability and robustness. We have assessed and collected data on the technical feasibility, validity, and safety of the designed SmartLac8 pump. This model is the first known mechatronic system for breast pumps that can simulate the infant oral mechanism in a user-controlled, interactive manner. The platform for a human study has been set up. A phase 1 clinical study will be conducted in collaboration with an academic research institution under IRB approval in the near future. Future work is planned for the integration of the PCB board, advanced control strategies, and additional pressure and temperature sensors to collect high-resolution vacuum pulse and accurate results.

## 7. Patents

A provisional patent has been filed and the device is under 5716.007 PRV: Powered breast pumps.

## Figures and Tables

**Table 1:** Comparison of the applied pressure from most commercially available breast pumps and breastfeeding infants in clinical experiments.

	References	Pump Brand	Vacuum Pressure	Compression Pressure
Breast-pumping	Evolve [24]	Evolve®Hygeia II	−23.331 to −37.730 kPa	-
	Freestyle [25]	Freestyle®Medela AG	−18.665 to −32.664 kPa	-
	Symphony [26,27]	Symphony®Medela AG	−26.397 to −42.530 kPa	-
	**References**	**Infant Age**	**Vacuum Pressure**	**Compression Pressure**
Breast-feeding	Prieto et al. [28]	6 Days to 7 months	−6.76 to −25.97 kPa	-
	Geddes et al. [29]	3 to 24 weeks	−5.991 to 19.3317	-
	Alatalo et al. [7]	6 Days to 21 months	−4.865 to −20.146 kPa	8.74 to 16.88 kPa

**Table 2:** Soft robotic pad design summary and dimensions.

Entity	Unit	Allowance
Actuator number	8	-
Air chamber number on each actuator finger	4	−
Soft Robotic Pad Diameter (mm)	100	± 2.5
Air chamber width (mm)	5	± 0.5
Air chamber height (mm)	5	0 ± 0.5
Air chamber length (mm)	7.5	± 0.5
Wall thickness (mm)	3	± 0.2

**Table 3:** Fitted parameters for open-loop system identification.

System ID	Stage 1		Stage 2	
	Vacuum	Compression	Vacuum	Compression
$\left[a_{1}^{0},a_{2}^{1}\right]$	[−0.378,−0.493]	[−0.844,−0.084]	[−0.574,−0.271]	[−1.1615,0.4471]
$\left[b_{1}^{0},b_{2}^{1}\right]$	[0.094,0.103]	[0.134,−0.067]	[−0.109,0.231]	[0.159,0.096]
RMSE	2.3721	12.363	3.5607	5.607
Goodness of Fit	91.77%	68.8%	74.88%	72.45%

**Table 4:** PID parameters and system performance.

Parameters	Vacuum Control	Compression Control
Proportional, P	0.011	167.3
Integration, I	0.109	334.6
Derivative, D	0.0157	-
Settling time, s	0.72	0.85
Overshoot	5.65%	7.64%
Steady state error	6.85%	10.03%
RMSE for Stage 1, kPa	2.3727	1.6509
RMSE for Stage 2, kPa	3.6544	3.1405

## Data Availability

Not applicable.

## Abbreviations

The following abbreviations are used in this manuscript:

3D	Three dimensional
MIMO	Multiple-input multiple-output
PID	Proportional-integral-derivative
RMSE	Root mean square error
ID	Identification
PC	Personal computer
